# HIF-1α Directly Controls WNT7A Expression During Myogenesis

**DOI:** 10.3389/fcell.2020.593508

**Published:** 2020-11-11

**Authors:** Federica Cirillo, Giulia Resmini, Elia Angelino, Michele Ferrara, Adriana Tarantino, Marco Piccoli, Paola Rota, Andrea Ghiroldi, Michelle M. Monasky, Giuseppe Ciconte, Carlo Pappone, Andrea Graziani, Luigi Anastasia

**Affiliations:** ^1^Laboratory of Stem Cells for Tissue Engineering, IRCCS Policlinico San Donato, San Donato Milanese, Italy; ^2^Department of Molecular Biotechnology and Health Sciences, University of Turin, Turin, Italy; ^3^Division of Genetics and Cell Biology, Chromatin Dynamics Unit, IRCCS San Raffaele Scientific Institute, Milan, Italy; ^4^Arrhythmology Department, IRCCS Policlinico San Donato, Milan, Italy; ^5^Department of Biomedical, Surgical and Dental Sciences, University of Milan, Milan, Italy; ^6^Vita-Salute San Raffaele University, Faculty of Medicine, Milan, Italy

**Keywords:** Hypoxia-inducible factor-1α, WNT7a, myogenesis, hypertrophy, Prolyl-hydroxylases, FG-4592

## Abstract

Herein we unveil that Hypoxia-inducible factor-1α (HIF-1α) directly regulates *WNT7A* expression during myogenesis. In fact, chromatin immunoprecipitation (ChiP) and site-directed mutagenesis experiments revealed two distinct hypoxia response elements (HREs) that are specific HIF-1α binding sites on the *WNT7A* promoter. Remarkably, a pharmacological activation of HIF-1α induced *WNT7A* expression and enhanced muscle differentiation. On the other hand, silencing of *WNT7A* using CRISPR/Cas9 genome editing blocked the effects of HIF-1α activation on myogenesis. Finally, treatment with prolyl hydroxylases (PHDs) inhibitors improved muscle regeneration *in vitro* and *in vivo* in a cardiotoxin (CTX)-induced muscle injury mouse model, paving the way for further studies to test its efficacy on acute and chronic muscular pathologies.

## Introduction

Skeletal muscle regeneration is a complex process which occurs throughout the lifespan thanks to an adult stem cell reservoir present in the adult muscle. In particular, satellite cells (about 1% of the muscle cell population) consist of roughly a 9:1 mixture of both Pax7^+^/Myf5^+^ muscle-committed progenitors, which can be triggered to differentiate and regenerate the tissue, and Pax7^+^/Myf5^–^ uncommitted *bona fide* adult stem cells, which keep replenishing the committed pool (Kuang et al., 2007). Many factors, including aging, can reduce or damage this stem cell population, thus altering muscle homeostasis and causing degeneration (Garcia-Prat et al., 2013). Unfortunately, previous attempts to increase regeneration by inducing muscle differentiation were unsuccessful, as they triggered a rapid depletion of the stem cell reservoir (Ambrosio et al., 2009). On the other hand, the cell-secreted factor Wnt7a has been shown to stimulate symmetric satellite stem cell division, dramatically enhancing muscle regeneration, whereas its depletion caused a marked reduction in the number of satellite cells following muscle healing (Le Grand et al., 2009). In fact, Wnt7a is a member of the Wnt signaling pathway, which is crucial during embryonic development and tissue homeostasis (Gough, 2012). In particular, Wnt7a has been shown to be essential for muscle differentiation and it promotes skeletal muscle hypertrophy (Tanaka et al., 2011). Indeed, Wnt7a overexpression, or treatment with a Wnt7a recombinant protein, has been shown to stimulate myoblasts to form hypertrophic myotubes (Tanaka et al., 2011; von Maltzahn et al., 2011; Bentzinger et al., 2014). Moreover, treatment with exogenous Wnt7a can affect myogenesis, as it increases satellite cells motility and engraftment, resulting in an enhancement of muscle strength (Bentzinger et al., 2014). However, people have struggled to therapeutically exploit Wnt7a and, to date, little is known about its upstream regulators to possibly envision other strategies to activate myogenesis (von Maltzahn et al., 2011). However, in this context, we previously reported that subjecting murine skeletal myoblasts C2C12 to a hypoxic pre-conditioning (1% O_2_ for 24 h) induced the upregulation of the hypoxia-inducible factor-1α (HIF-1α) as well as an activation of the non-canonical WNT-pathway, including Wnt7a, eventually leading to an activation of myogenesis and the formation of hypertrophic myotubes (Cirillo et al., 2017). However, we failed to recognize the mechanism of the observed effects.

Thus, in this study, we investigated the possible mechanistic link between the HIF-1α and *WNT7A*. In particular, chromatin immunoprecipitation (ChiP) and site-directed mutagenesis experiments revealed that HIF-1α specifically binds to two distinct, and previously unreported, hypoxia response elements (HREs) on the *WNT7A* promoter, eventually activating its transcription and inducing myogenesis.

## Materials and Methods

### Cell Cultures

C2C12 murine myoblasts (Merck), HIF-1α-silenced C2C12 murine myoblasts (shHIF-1α), and *WNT7A-KO* silenced C2C12 murine myoblasts, which were obtained in our previous study (Cirillo et al., 2017), were cultured in growth medium (GM) made of Dulbecco’s modified Eagle’s medium (DMEM, Merck) with high glucose concentration (4.5 g/L) supplemented with 10% (v/v) fetal bovine serum (FBS, Merck), 2 mM glutamine (Merck), penicillin/streptomycin 1X (Euroclone; GM) at 37°C in a 5% CO_2_ and 95% air-humidified atmosphere. Cells were treated for 24 h under hypoxic culture conditions (1% O_2_) or with prolyl-hydroxylases (PHDs) inhibitors (IOX2 or FG-4592) at 37°C in a 5% CO_2_/21% O_2_ atmosphere. In order to induce muscle differentiation, 75 × 10^3^ C2C12 murine myoblasts were seeded in 35 mm culture plates, cultured in GM for 24 h, and then switched to a differentiation medium (DM) containing DMEM supplemented with 2% (v/v) horse serum (HS; Merck; Scaringi et al., 2013). The DM was changed every day for 7 days. Cells were analyzed at 24 h post-treatment (PT) and at the third and seventh days of skeletal muscle differentiation. These cells were tested and found to be negative for mycoplasma contamination before the experiments.

### WNT7A Plasmid Construct Preparation

Genomic DNA was extracted from C2C12 murine myoblasts and used as the template for *WNT7A* promoter amplification. The PCR reaction was prepared using a Phusion High-Fidelity kit (New England Biolabs) and employing the following primers: forward 5′-GGGGTACCCCGGAAGCTATCACAGGCTT-3′, containing *Kpn*I enzymatic site, and reverse 5′-GGGT TCGAACCCTAGCATCCTTCGCTAACT-3′, containing the *Hin*dIII enzymatic site. The PCR fragment and the vector pNL1.1(Nluc; Promega) were digested with KnpI and *Hin*dIII (Promega) restriction enzymes for 2 h at 37°C, and then they were purified from agarose gel using Wizard^®^ SV Gel and PCR Clean-Up System (Promega). The fragment was inserted into the multiple cloning site of pNL1.1 vector using T4 Ligase Fast (Promega), and the reaction was incubated for 1 h at room temperature. The presence of the insert in the plasmid was finally verified by DNA sequencing.

### Nano-Glo Luciferase Reporter Assays

The regulation of the *WNT7A* promoter mediated by HIF-1α was evaluated with the Nano-Glo^®^ Dual-Luciferase^®^ Assay System kit (Promega), which enables a high-throughput analysis of mammalian cells containing the following reporter genes: (a) Nanoluc (pNL1.1empty and pNL1.1WNT7A_promoter) and (b) pGL4.54(luc2/TK), containing the reporter gene Firefly as internal control. Briefly, cells were seeded in a 96-well plate at a concentration of 5 × 10^3^ cells per well, transfected with the pNL1.1 or pNL1.1WNT7A_promoter (90 ng) plasmids and pGL4.54(luc2/TK; 10 ng) plasmid as internal control, according to ViaFect Transfection Reagent^®^ protocol (Promega), and then incubated for 24 h. After transfection, cells were exposed for 24 h to hypoxic culture conditions (1% O_2_) or to the PHDs inhibitors, IOX2 (Merck) or FG-4592 (Roxadustat, Selleckchem), at their respective IC_50_ working concentrations. Then, an equal volume of ONE-Glo^TM^ EX Luciferase Assay Reagent was added to each well, incubated for 30 min, and then the luminescence was analyzed with a Varioskan^TM^ LUX Multimode Microplate Reader (Thermo Fisher Scientific). To turn off the luminescence of the Firefly luciferase and provide the substrate for the Nanoluc, an equal volume of NanoDLR^TM^ Stop&Glo^®^ Reagent was added to the mixture and incubated for 30 min before analysis.

### Chromatin Immunoprecipitation Assays

The identification of the binding sequence of the HIF-1α protein on the *WNT7A* promoter was obtained with a ChIP using a SimpleChIP^®^ Enzymatic Chromatin IP Kit (Magnetic Beads, Cell Signaling Technology) according to the manufacturer’s instructions. Briefly, 1 × 10^7^ C2C12 cells were seeded in GM and cultured for 24 h under hypoxic (1% O_2_) or normoxic (21% O_2_) culture conditions. Then, cells were harvested by scraping and the genomic DNA was cross-linked with bound proteins with 1% formaldehyde for 10 min at room temperature. Then, the genomic DNA was digested with Micrococcal Nuclease for 20 min at 37°C to achieve DNA sizes ranging from 150 to 900 bp, and then subjected to sonication (Branson Sonifier 150). The immunoprecipitation was achieved using: (a) rabbit monoclonal anti-HIF-1α, 1:1,000 dilution (clone D2U3T, Cell Signaling Technologies), (b) rabbit monoclonal anti-Histone-H3 as technical positive control, 1:50 dilution (D2B12, Cell Signaling Technologies), and (c) IgG rabbit, 5 μg, as the negative control. The mixture was then incubated at 4°C overnight under rotation. The immuno-complexes were collected with ChIP Grade Protein G Magnetic Beads and incubated for 2 h at 4°C under rotation. The elutes were digested with Proteinase K at 65°C for 4 h, and the precipitated DNA was recovered using DNA Purification Spin Columns. Finally, the purified DNA was amplified by Real-Time PCR using the following ChIP primers designed on the *WNT7A* promoter and on the known *VEGF-HRE*: WNT7A1 forward 5′-GGAAGCTATCACAGGCT-3′ and reverse 5′-ACCGTGGATTCAAGGA-3′; WNT7A2 forward 5′- TGTCAGCATGGCTGGCGC-3′and reverse 5′-AGTTCTTCATGCATAC-3′; WNT7A3 forward 5′-GCAGTCT GGAAGGCTAAGC-3′ and reverse 5′-GTCAAACTCAAAGTC AC-3′; WNT7A4 forward 5′-TTCACCTCCTCAGTTTCTCCA-3′ and reverse 5′-AGCCCAATGACCAGTAGCAG-3′; WNT- 7A5 forward 5′-TCCAGTCCAATTCCAAGTC-3′ and reverse 5′-GTCTGTGCGAGGGGCTGA-3′; WNT7A6 forward 5′-GAG GGTGAGGAGAGAATG-3′ and reverse 5′-CCTTGTTAGAG CTCTGTCA-3′; WNT7A7 forward 5′-CGTGAGGGTTCTG AAATG-3′ and reverse 5′-GGAATCCTGCCTGCCTAG-3′; VEGF-HRE forward 5′-GAACAAGGGCCTCTGTCT-3′ and reverse 5′-CACCAAATTTGTGGCACT-3′.

### Site-Directed Mutagenesis

The identification of HIF-1α binding sites on the *WNT7A* promoter was performed by site-directed mutagenesis experiments followed by luciferase assays. Each HRE sequence, which was identified as a putative binding site for HIF-1α on the *WNT7A* promoter, was deleted using the QuickChange Lightning Site-Directed Mutagenesis kit (Agilent). Briefly, in order to generate a mutant plasmid containing a single HRE deletion, a thermal cycling reaction mix was performed using pNL1.1WNT7A_promoter as the template, together with two synthetic mutagenic primers suitably designed to insert the desired deletion. Following the temperature cycling, the product was treated with Dnp I endonuclease, which recognized both methylated and hemimethylated DNA, to digest the parental DNA template and to select the mutant DNA. The vector DNA containing the deletion was then transformed into XL10-Gold ultracompetent cells. Each mutant plasmid was subjected to DNA sequencing to confirm the correct deletion.

### Determination of the IC_50_ for the PHDs Inhibitors IOX2 and FG-4592

The IC_50_ was calculated using a Dual-Glo^®^ Luciferase Assay System kit (Promega) that enables a high-throughput analysis of mammalian cells containing the Firefly [Luciferase-pcDNA3, oxygen dependent domain (ODD)-Luciferase-pcDNA3] and Renilla pRenilla-Cytomegalovirus (pRL-CMV) luciferase reporters. Briefly, 5 × 10^3^ cells per well were seeded in a 96-well plate, and then transfected for 24 h with a Luciferase-pcDNA3 or an ODD-Luciferase-pcDNA3 (80 ng), containing the firefly gene reporter. A co-transfection with pRL-CMV (8 ng), containing the Renilla gene reporter, was performed as an internal control, according to the ViaFect Transfection Reagent^®^ protocol (Promega). After transfection, cells were treated with 10, 25, 50, and 100 μM IOX2 or FG-4592 (Roxadustat) for 24 h, and then an equal volume of Dual-Glo Luciferase Reagent was added to each well, incubated at room temperature for 30 min, and then analyzed with a Varioskan^TM^ LUX Multimode Microplate Reader (Thermo Fisher Scientific). Then, an equal volume of Dual-Glo^®^ Stop & Glo^®^ Reagent was added to each well in order to quench the luminescence from the firefly reaction and to provide the substrate for the Renilla luciferase. The mixture was incubated for 30 min before analysis. The IC_50_ values were calculated by linear regression.

### Cell Viability Assays

RealTime-Glo^TM^ kits MT Cell Viability Assay (Promega) was used to investigate the effects of PHDs treatment or *WNT7A* silencing on cell viability. Briefly, 2 × 10^3^ C2C12 and *WNT7A*-KO murine myoblasts, treated with 50 μM IOX2 or 40 μM FG-4592, were seeded in a 96-well plate and incubated for 3, 6, 24, and 48 h (T0) with MT Cell Viability Substrate (1:1,000 dilution) and NanoLuc^®^ Enzyme (1:1,000 dilution). The luminescence signal was measured by Varioskan^TM^ Lux Multimode Microplate Reader.

### Cell Toxicity Assays

To assess the cytotoxicity of the PHDs inhibitors, 5 × 10^3^ C2C12 murine myoblasts were seeded in a 96-well plate and treated with 50 μM IOX2 or 40 μM FG-4592 for 24 h before analysis with a CellTox^TM^ Cytotoxicity Assay (Promega). Briefly, 100 μl of CellTox Buffer containing CellTox Green Dye (1:500 dilution) was added to each well and incubated at room temperature for 15 min. The fluorescence analysis (excitation wavelength of 485-500 nm and emission filter of 520-530 nm) was measured by Varioskan^TM^ Lux Multimode Microplate Reader.

### RNA Extraction and Gene Expression by Real-Time Quantitative PCR (qPCR)

Total RNA was isolated using ReliaPrep^TM^ RNA Miniprep Systems (Promega), and reverse transcribed to cDNA using the iScript cDNA synthesis kit (BioRad), according to the manufacturer’s instructions. Real-Time Quantitative PCR was performed on 10 ng of cDNA template, 0.2 μM primers, and GoTaq^®^ qPCR Master Mix (Promega) in 20 μl final volume using a StepOnePlus^®^ Real-Time PCR System (Applied Biosystem). The following primers were used: VEGF forward 5′-AAAAACGAAAGCGCA-3′ and reverse 5′-TTTCTCCGCTCTGAA-3′; PHD2 forward 5′-CTGT GGAACAGCCCTTTTT-3′ and reverse 5′- CGAGTCTCTCTG CGAATCCT-3′; MYOG forward 5′-AGCCACACTGAGGGA-3′ and reverse 5′-GTTGAGGGAGCTGAG-3′; myosin heavy chain (MHC) forward 5′-TGGAGCAGGAGGAATACAAG-3′ and reverse 5′-GCATAGTGGATGAGGGAGAA-3′; WNT1 forward 5′-AACCTTCACAACAACGAG-3′ and reverse 5′-GTTGCTGCCTCGGTTG-3′; WNT3A forward 5′-CTTAGTG CTCTGCAGCCTGA-3′ AND REVERSE 5′-GAGTGCTCAGA GAGGAGTACTGG-3′; WNT4 forward 5′-GGGTGGAGTGCA AGTGTCA-3′ and reverse 5′-CACGCCAGCACGTCTTTAC-3′; WNT7A forward 5′-ACTGTGGCTGCGACAAG-3′ and reverse 5′-CTTCATGTTCTCCTCCAG-3′; WNT9a forward 5′-TCGTGGGTGTGAAGGTGATA-3′ and reverse 5′-CAGG AGCCAGACACACCAT-3′; WNT11 forward 5′-CAGGATCCC AAGCCAATAAA3′ and reverse 5′-TCCAGGGAGGCACGT AGA-3′; MYOR forward 5′-GCCCAGCGACATTTCTTC-3′ and reverse 5′-CGCTTCCTCTTGCATCCT-3′; RPL13 forward 5′- CTCGGCCGTTCCTGTAT-3′ and reverse 5′-GTGGAAGTGGGGCTTCAGTA-3′. RPL13 was used as the housekeeping gene. The amplification program consisted of an initial denaturation at 95°C for three min, followed by 40 cycles of 5 s each at 95°C and 30 s at 57°C; WNT7A was amplified at 53°C. Relative quantification of target genes was performed in triplicate and calculated by the equation 2^–ΔΔ^ ^Ct^.

### Total Protein Extraction and Isolation of the Nucleus Compartment

For total protein extraction, 7.5 × 10^4^ cells were seeded and harvested at the seventh day of skeletal muscle differentiation, as previously described. Cells were collected after enzymatic digestion with trypsin (Merck), lysed by sonication, and centrifuged at 800 × for 10 min. Supernatant was used to determine total protein content with the bicinchoninic acid (BCA) Protein Assay Kit (Pierce), according to the manufacturer’s instructions.

For nuclei separation, 5 × 10^6^ cells were collected after enzymatic digestion with trypsin (Merck), centrifuged at the maximum speed, resuspended in 400 μl of Buffer A (10 mM KCl, 0.1 mM Ethylenediaminetetraacetic acid (EDTA), 1 mM Dithiothreitol (DTT), 10 mM Hepes, pH 7.9), containing protease and phosphatase inhibitors cocktail (Merck), and incubated on ice for 20 min. Then, 10% NP40 was added to the cell suspension, mixed for 40 s, and centrifuged 40 s at the maximum speed. After centrifugation, the pellet was resuspended in 80 μl of Buffer C (0.4 M NaCl, 1 mM EDTA, 1 mM DTT, 20 mM Hepes, pH 7.9), containing a protease and phosphatase inhibitors cocktail, and then mixed for 30 min at 4°C at the maximum speed. Finally, the nuclear suspension was centrifuged for 5 min at 4°C at the maximum speed, collected and transferred to a new tube. The total protein content was determined with the BCA Protein Assay Kit (Pierce), according to the manufacturer’s instructions.

### Western Blot Analyses

Proteins were denatured by boiling for 10 min in sample buffer (0.6 g/100 mL Tris, 2 g/100 mL SDS, 10% glycerol, 1% 2-mercaptoethanol, pH 6.8) and loaded into a 10% SDS–PAGE gel, then transferred onto a nitrocellulose membrane (Trans-blot, Bio-Rad Laboratories) by electroblotting. Nitrocellulose membranes were incubated with a blocking solution containing 5% (w/v) non-fat dry milk or 5% (w/v) BSA (Merck) in Tris-buffer saline with 0.1% Tween^®^ 20 (TBS-T) for 1 h. Blots were incubated for 2 h at room temperature with the following primary antibodies: rabbit monoclonal anti-HIF-1α, 1:1,000 dilution (clone D2U3T, Cell Signaling Technology), rabbit monoclonal anti-HIF2α, 1:250 dilution (clone D9E3, Cell Signaling Technology) goat polyclonal anti-Lamin A/C, 1:1,000 dilution (clone N-18, Santa Cruz Biotechnology), mouse monoclonal anti-MyoD1, 1:1,000 dilution (clone 5.2F, Abcam), mouse monoclonal anti-myosin (skeletal, fast), 1:1,000 dilution (clone MY-32, Merck), rabbit polyclonal anti-Wnt7a, 1:1,000 dilution (abcam) and rabbit polyclonal anti-EE1a, 1:1,000 dilution (Cell Signaling Technology). The total amount of transferred proteins was used to normalize mice proteins using the REVERT Total Protein Stain kit (LI-COR Biotechnology), following manufacturer’s instructions. Membranes were washed three times for 10 min with TBS-T, and then incubated with the appropriate anti-mouse, anti-rabbit, or anti-goat HRP-conjugated secondary antibodies (Dako, Agilent Technologies), 1:2,000 dilution, for 1 h at room temperature. After three washes for 10 min with TBS-T, the immunoreactive bands were visualized using the enhanced chemiluminescence detection kit reagents (ECL Advance, GeHealthcare), according to the manufacturer’s instructions.

### Immunofluorescence Staining

After differentiation for 7 days in DM, cells were washed with phosphate saline buffer (PBS) and fixed for 15 min in 4% (w/v) paraformaldehyde at room temperature. For permeabilization and blocking, cells were incubated for 1 h in the presence of PBS 0.1% (v/v) Triton X-100 (TX-100, Merck) and 5% (w/v) FBS (Merck) at room temperature. Then, cells were incubated for 2 h at room temperature with mouse monoclonal anti-Myosin (Skeletal, fast; clone MY-32, Merck), diluted 1:200 in PBS 0.1% (v/v) Triton X-100 (TX-100) and 5% (w/v) FBS. After incubation, cells were washed three times in PBS and incubated for 1 h at room temperature with an anti-mouse FITC-conjugated secondary antibody (Jackson ImmunoResearch), diluted 1:200. Cell nuclei were counterstained with Hoechst 33,342 (1:500 dilution, Merck). Myogenesis was assessed by measuring the myotube area and the number of myonuclei per myotube using a fluorescent microscope (Olympus TH4-200) equipped with an acquisition camera. To quantify both differentiation and fusion indexes, 10 fields were chosen randomly and, for each field, a minimum of one hundred myosin-positive myotubes with more than two myonuclei were measured using the ImageJ v1.49o software. The area and the number of nuclei per myotube was the mean of ten measurements averaged from three different experiments. The negative control of MHC was performed using proliferating wild-type- and *WNT7A*-KO-silenced murine myoblasts.

### CRISPR/Cas9 Mediated Knockout of the *WNT7A* Gene in C2C12 Murine Myoblasts

The knockout of the *WNT7A* gene was obtained performing CRISPR/Cas9 genome editing. In particular, crRNAs and tracrRNA were obtained from TrueGuide^TM^ Synthetic gRNA kit (Thermo Fisher Scientific), and they were reconstituted and annealed following the manufacturer’s instructions. In particular, the target sequence 5′-GGGCATAGTCTACCTCCGGATCGG-3′ was selected as the crRNA of the *WNT7A* gene, while the target sequence 5′-AAAUGUGAGAUCAGAGUAAU-3′, which doesn’t recognize any sequence in the human genome (Thermo Fisher Scientific), was used as the negative control. Briefly, they were re-suspended using 1X Tris-EDTA buffer pH 8.0 to prepare a 100 μM stock solution. Then, gRNAs were generated preparing a mix composed of 10 μL crRNA, 10 μL tracrRNA, 10 μL annealing buffer, and 20 μL nuclease-free water. The mixture was incubated at 95°C for 5 min followed by 10 min on 78°C, and then 25°C for 5 min.

The transfection was performed seeding 5 × 10^5^ C2C12 murine myoblasts in a 24-well plate. Two different mixtures were prepared:

• 1,250 ng of TrueCut^TM^ Cas9 Protein v2 (Thermo Fisher Scientific), 2.5 μl of Lipofectamine^TM^ Cas9 Plus^TM^ Reagent, 240 ng of gRNA, and 25 μl of Opti-MEM I Medium.
• 1.5 μl Lipofectamine^TM^ CRISPRMAX^TM^ reagent (ThermoFisher Scientific) and 25 μl of Opti-MEM I Medium.

The diluted Lipofectamine^TM^ CRISPRMAX^TM^ reagent in Opti-MEM I Medium was incubated for 1 min at room temperature, then added to gRNA/Opti-MEM I solution for 15 min. Then, the mixture was added to murine myoblasts and incubated at 37°C for 2 days. After the incubation, single-cell clones were isolated using a limiting dilution cloning in 96-well plates, following the manufacturer’s instructions. The efficiency of *WNT7A* knockout (*WNT7A*-KO) was verified by sequencing.

### Genomic DNA Extraction and Amplification by PCR

Genomic DNA was isolated using Wizard^®^ Genomic DNA Purification kit (Promega), according to the manufacturer’s instructions, and used to amplify the *WNT7A* and *RPL13* genes using the following primers: *WNT7A* forward 5′-CTTGTTGCGCTTGTTCTCC-3′ and reverse 5′-CGCAATTCCACAGACTCG-3′; *RPLI* 5′-CTCGGCCGTTC CTGTAT-3′ and reverse 5′-GTGGAAGTGGGGCTTCAGTA-3′. The amplification program consisted of an initial denaturation at 98°C for 30 s, followed by 30 cycles of 10 s each at 98°C, 15 s at 57°C, and 30 s at 72°C. The amplification was concluded by a final extension step at 72°C for 10 min.

### Animals

The procedure involving mice was performed according to the animal protocol guidelines described by the Institutional Animal Care and Use Committee (IACUC) authorization no. 89-2018-PR at San Raffaele Scientific Institute (Milan, Italy). All mice were housed for two weeks in individual cages with a 12-h light/dark cycle, allowing free access to food and water. All efforts were made to minimize animal suffering and to reduce the number of mice used, in accordance with the European Communities Council Directive of November 24, 1986 (86/609/EEC). The number of mice estimated sufficient to detect a difference between two means as large as one SD unit with 80% power and a significance level of 95% at Student’s *t*-test were calculated with the program by R.V. Lenth^1^ and no formal randomization procedure was used. The investigators conducting the experiments were blind to the experimental group assessed. The investigators quantifying the experimental outcomes continued to be blinded to the animal group or intervention. Finally, the statistical evaluation of the experimental data was performed by another investigator who was not directly involved in data collection and parameter measurement.

### Cardiotoxin-Induced Muscle Regeneration and Exogenous FG-4592 Administration

Experiments on muscle regeneration were conducted on 8–10-week-old male C57Bl/6N mice, matched for weight, purchased from Charles River Laboratories (Calco, Italy). Cardiotoxin (CTX, from Naja mossambica mossambica, Latoxan, Portes-les-Valence, France^2^) was dissolved in sterile saline to a final concentration of 10 μM. Mice were anesthetized by isoflurane inhalation, and hindlimbs were shaved and cleaned with alcohol. Tibialis anterior (TA) muscles were injected with 45 μl of CTX with a 30-gauge needle, with 15 micro-injections of 3 μl CTX each in the mid-belly of the muscle to induce homogeneous damage. The TA muscles of the contralateral hindlimbs were injected with a saline solution. A 50 mg/ml of FG-4592 stock solution was first prepared in DMSO, then further diluted in sterile PBS to 1 mg/ml and stored aliquoted at −20°C. FG-4592 was administrated 24 h before CTX injury by i.p. injection using 31-gauge needles at a dose of 10 mg/kg (Hoppe et al., 2016; Xuan et al., 2018). DMSO, diluted at 2% in saline solution, was inoculated by i.p. injection to control mice. The experiments were conducted using seven mice for each group, although one mouse of the FG-4592 group died spontaneously during the procedure. Mice were sacrificed 7 days after CTX treatment.

### Histological Analyses

Histological analysis was performed as previously reported (Reano et al., 2017). Briefly, TA muscles were frozen in liquid nitrogen-cooled isopentane and mounted in Killik embedding medium (Bio-optica). Transverse muscle sections (7 μm) were cryosectioned from the mid-belly of each muscle. Sections were stained with Hematoxylin (Bio-optica)/Eosin (Merck) to reveal general muscle architecture. For immunofluorescence, after fixing in PFA 4% for 10 min, slices were permeabilized with 0.2% Triton X-100 in 1% BSA for 15 min and blocked with 4% BSA for 1 h. The primary antibodies anti-laminin (1:200; Dako, Agilent Technologies) and anti-CD31 (1:100; Space srl) were incubated overnight at 4°C, while the incubation with the secondary antibody (1:450, anti-rabbit, Alexa Fluor^TM^ Antibodies) was performed at room temperature for 1 h. Finally, the slices were incubated with Hoechst 33342 (Merck) for 15 min. Images were acquired using Axio Lab.A1 (Zeiss) and quantified with ImageJ v1.49o software.

### Statistical Analyses

All assays were performed from three up to eleven replicates, and the quantitative data are displayed as mean ± standard deviation. The statistical analysis was performed with GraphPad Prism 7.0 (GraphPad Software, United States). The Student’s *t*-test or One-Way ANOVA and Dunnett test for multiple comparisons were used to determine the significance values. *P* values of less than 0.05 were considered to be significant. All *P* values were calculated from data obtained from at least three independent experiments. Statistical significance was assumed for ^∗^*p* < 0.05. All error bars represent the standard deviation of the mean.

## Results

### HIF-1α Binding on the *WNT7A* Promoter

**FIGURE 1:**
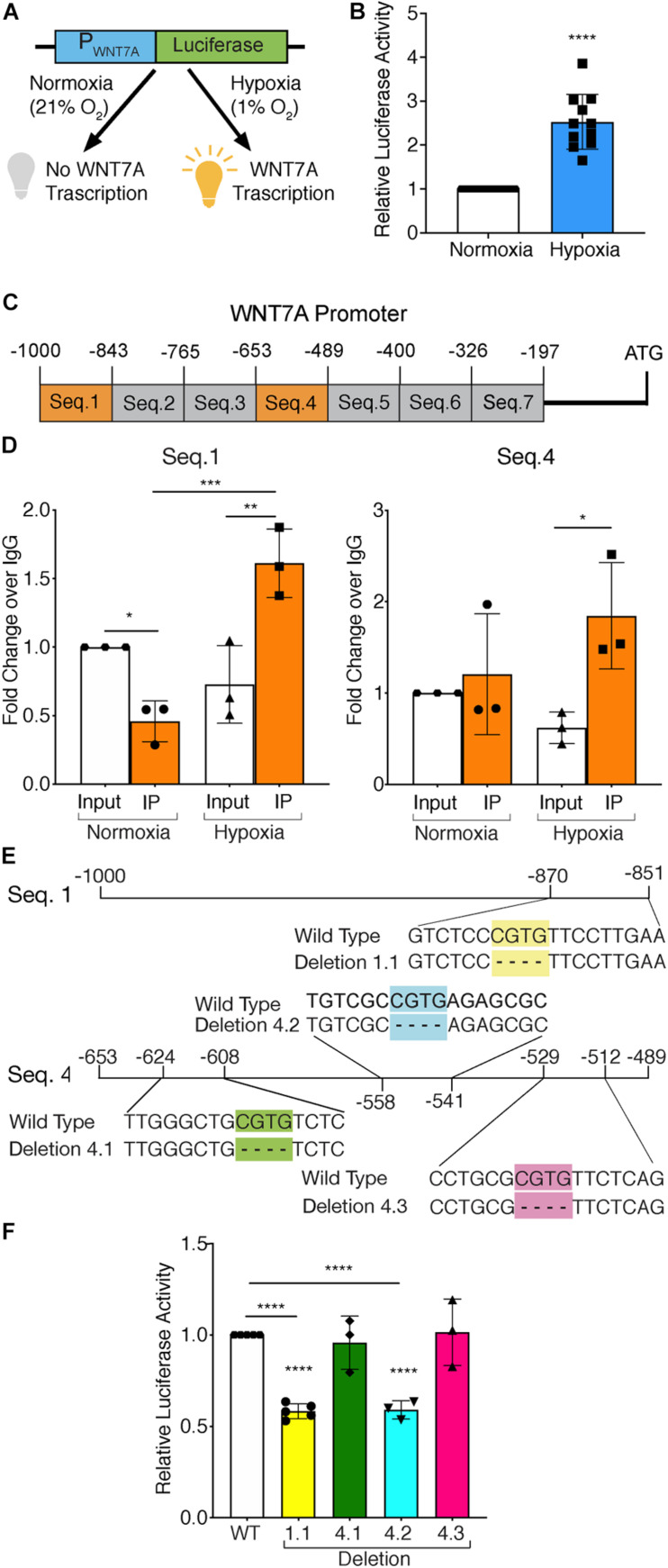
Assessment of hypoxia inducible factor-1α (HIF-1α) binding to the *WNT7A* promoter after 24h at 1% O_2_. **(A)** Schematic representation of pNL1.1 plasmid containing the *WNT7A* promoter upstream of the luciferase gene reporter. Under normoxia, HIF-1α is degraded and cannot bind the HRE sequences on the *WNT7A* promoter, inhibiting the luminescence signal; under hypoxia, HIF-1α is stabilized and can bind the *WNT7A* promoter, inducing the expression of the luciferase gene. **(B)** Quantification of pNL1.1-*WNT7A* promoter activity by luciferase assay in murine myoblasts under normoxia and hypoxia (1% O_2_; *n* = 11). **(C)** Schematic representation of the seven binding sequences designed within the *WNT7A* promoter and used for qPCR analysis after chromatin immunoprecipitation (ChiP). **(D)** qPCR analysis of HIF-1α binding on Seq.1 and Seq.4 of the *WNT7A* promoter (*n* = 3). **(E)** Schematic representation of the deletions of the HRE binding sequences identified in Seq. 1 (Deletion 1.1) and Seq. 4 (Deletion 4.1, 4.2, and 4.3) inserted in the pNL1.1-*WNT7A* promoter construct using site-directed mutagenesis experiments. **(F)** Quantification of mutated pNL1.1-*WNT7A* promoter activity by luciferase assay (*n* = 5 for mutation 1.1, *n* = 3 for mutation 4.1, 4.2 and 4.3). Data information: all data represent mean ± SD. *p < 0.05, **p < 0.01, ***p < 0.001, ****p < 0.0001 (Ordinary one-way ANOVA).

To test whether HIF-1α activation was directly responsible for *WNT7A* transcriptional induction, HIF-1α-silenced (kd-HIF-1α) and control (HIF-1α) murine myoblasts were seeded to set up a luciferase assay. They were transiently co-transfected with plasmid pNL1.1WNT7A_promoter, containing the luciferase gene under the control of the *WNT7A* promoter (Figure 1A), and with plasmid pGL4.54 (luc2/TK), containing the luciferase gene under the TK promoter activity as the internal control. After transfection, cells were switched to hypoxic culture conditions (1% O_2_) for 24 h, then tested for *WNT7A* promoter activation by luciferase assay. Results revealed a 2.5-fold luminescence increase in WT myoblasts upon the hypoxic treatment, as compared to normoxic controls, which was consistent with an enhancement of the *WNT7A* promoter activity under hypoxia (Figure 1B). On the other hand, a 40% silencing of HIF-1α nuclear translocation, tested by WB, (Supplementary Figures S1A,B) caused a significant reduction (-40% luminescence) in *WNT7A* promoter activation under hypoxia, as compared to WT myoblasts (Supplementary Figures S1C,D). Next, quantitative ChIP (qChIP) assays were performed to identify any binding regions of HIF-1α on the *WNT7A* promoter, by dividing it into seven putative binding sequences from −1,000 to −197 bp, each containing at least one Hypoxia Responsive Element (HRE; 5′-CGTG-3′; Figure 1C). ChIP for HIF-1α binding to the *VEGF-HRE* was included as a positive control (Supplementary Figure S2A). After qChIP with the HIF-1α antibody, the *VEGF-HRE* and each sequence of *WNT7A* promoters were amplified by qPCR with specific primers. Results confirmed the specificity of HIF-1α binding on *VEGF-HRE* upon hypoxia pre-conditioning (Supplementary Figure S2B). Moreover, while no significant binding on *WNT7A* promoter was observed under normoxia, results showed that, under hypoxia, HIF-1α bound to Seq.1 (−1,000 −843 bp) and Seq.4 (−653 −489 bp; Figure 1D and Supplementary Figure S3), which contain one and three HRE sequences, respectively (Figure 1E). To further discriminate among these four HRE sites, which were responsible for HIF-1α binding, site-directed mutagenesis experiments were performed to generate four mutants of the *WNT7A* promoter (1.1, 4.1, 4.2, and 4.3), each carrying a single HRE deletion (Figure 1E). C2C12 murine myoblasts were transiently co-transfected with plasmid pNL1.1-WNT7A (Wild Type or one of the mutants) and with plasmid pGL4.54(luc2/TK), as the internal control, and then cultured under hypoxic conditions (1% O_2_) for 24 h. Luciferase assays revealed a 40% decrease in luminescence for deletion 1.1 and 4.2, while no significant differences could be observed for mutants 4.1 and 4.3, compared to WT. This data supports the hypothesis that only HREs 1.1 and 4.2, localized on sequence 1 and 4, respectively, were responsible for HIF-1α binding on the *WNT7A* promoter (Figure 1F). Then, to assess the time course of Wnt7a protein accumulation, murine myoblasts were cultured for 3, 6, 12, and 24 h under normoxic or hypoxic culture conditions. Results revealed no significant changes in Wnt7a protein under normoxia, but a significant increase of 1.6 and 1.7 folds at 12 and 24 h, respectively, under hypoxia (Supplementary Figures S4A,B). Finally, to exclude HIF-2α involvement in the activation of *WNT7A* promoter, its nuclear translocation was evaluated in murine myoblasts pre-treated under hypoxia (1% O_2_) for 24 h. Results showed no significant alterations in HIF-2α accumulation into the nuclei (Supplementary Figures S5A,B).

### The Pharmacological Activation of HIF-1α Induces *WNT7A* Transcription

**FIGURE 2:**
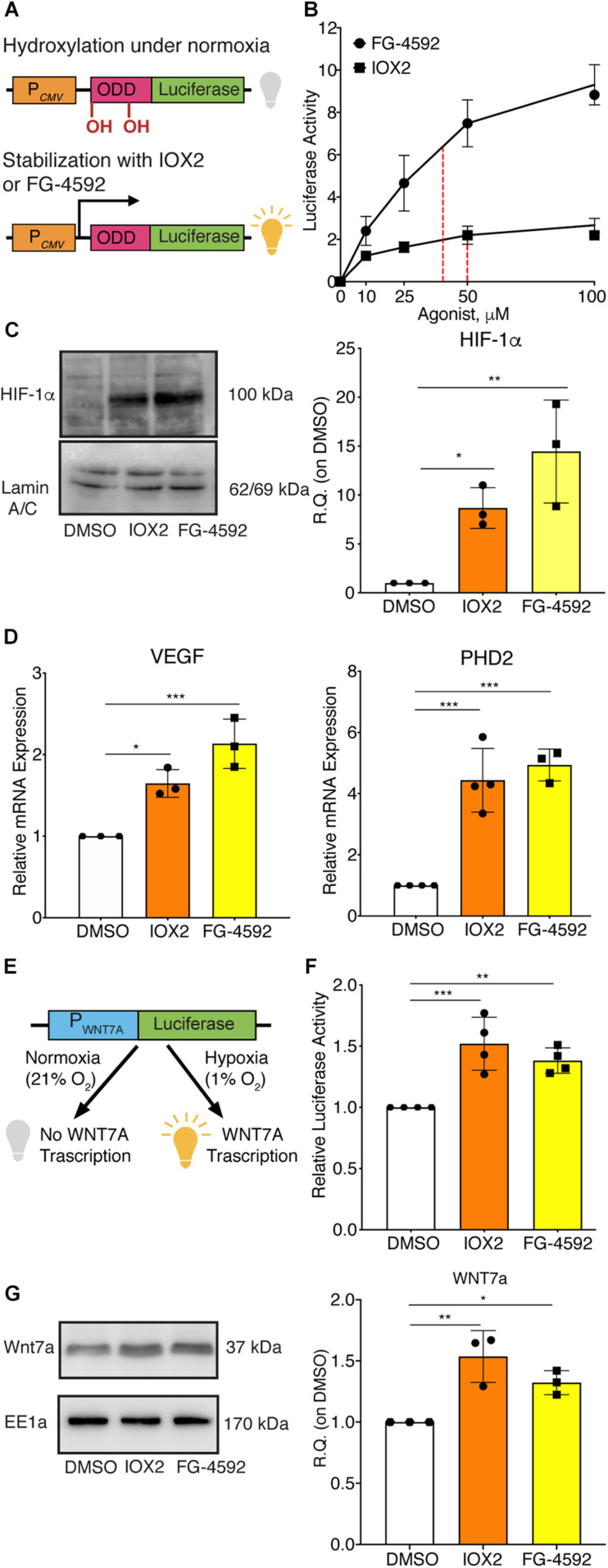
Pharmacological activation of hypoxia inducible factor-1α (HIF-1α) with prolyl-hydroxylases (PHDs) inhibitors and its effects on *WNT7A* regulation. **(A)** Schematic representation of the pcDNA3 construct containing the oxygen dependent domain (ODD)-luciferase reporter gene. Under normoxia, the proline residues on the ODD sequence are hydroxylated by the PHDs, and the complex degraded by the proteasome, eventually inhibiting the generation of the luminescence signal; the pharmacological inhibition of the PHDs causes the stabilization of the ODD, inducing the production of luminescence. **(B)** IC_50_ quantification of IOX2 or FG-4592 by luciferase assay with the ODD-pcDNA3 construct (*n* = 3). **(C)** Western blot analysis and relative quantification of HIF-1α nuclear localization in murine myoblasts treated with IOX2 or FG-4592 for 24 h. The nuclear marker Lamin A/C was used as the housekeeper (*n* = 3). **(D)** qPCR analysis of HIF-1α target genes, *VEGF* and *PHD2*, upon PHDs inhibition with IOX2 or FG-4592 (*n* = 3). **(E)** Schematic representation of pNL1.1 plasmid containing the *WNT7A* promoter upstream of the luciferase gene reporter under normoxia and upon IOX2 or FG-4592 treatment. **(F)** Quantification of pNL1.1-*WNT7A* promoter activity by luciferase assay of murine myoblasts treated with IOX2 or FG-4592 (*n* = 4). **(G)** Western blot analysis and relative quantification of Wnt7a accumulation in murine myoblasts treated with IOX2 or FG-4592 for 24 h. EE1a was used as the housekeeper (*n* = 3). Data information: all data represent mean ± SD. *p < 0.05, **p < 0.01, ***p < 0.001 (Ordinary one-way ANOVA).

Since HIF-1α can directly activate *WNT7A* transcription, it was assessed whether the induction of *WNT7A* could be also obtained through the pharmacological stabilization of HIF-1α using the two PHDs inhibitors, IOX2 and FG-4592 (Maxwell and Eckardt, 2016). For this purpose, the IC_50_ of both compounds was determined by transiently co-transfecting C2C12 myoblasts with the ODD-luciferase-pcDNA3 plasmid, containing the oxygen dependent domain (ODD) of HIF-1α, and with the pRL-CMV plasmid, as the internal control (Figure 2A). Then, transfected cells were treated for 24 h with IOX2 or FG-4592 at different concentrations (10, 25, 50, and 100 μM) under normoxic conditions, and the emitted luminescence was collated to untreated controls. Results showed an IC_50_ of 50 μM and 40 μM for IOX2 or FG-4592, respectively (Figure 2B). Next, murine myoblasts were cultured with IOX2 and FG-4592 at their IC_50_ concentration for 3, 6, 12, 24, and 48 h and the effects on proliferation rate were analyzed. Results revealed that pre-treatment with the PHDs inhibitors did not significantly modify the cell growth (Supplementary Figure S6A). Furthermore, a 24 h pre-conditioning with the drugs did not induce any toxic effect on murine myoblast (Supplementary Figure S6B). To assess the activation of HIF-1α pathway, its nuclear translocation and the expression of its main target genes were evaluated. Unlike the untreated control, both IOX2 and FG-4592 treatments induced HIF-1α nuclear translocation, with an 8.8- and 15-fold protein increase, respectively (Figure 2C). Moreover, qPCR analyses of HIF-1α target genes demonstrated that IOX2 treatment induced a 1.6- and 4.4-fold increase in the expression of *VEGF* and *PHD2*, respectively, while FG-4592 promoted a 2.1- and 4.8-fold increase of the same genes, as compared to untreated controls (Figure 2D). Successively, the activation of the *WNT7A* promoter was investigated by luciferase assay, revealing that IOX2 and FG-4592 treatments induced a 1.4- and 1.3-fold luminescence increase, respectively (Figures 2E,F). To further confirm the activation of the *WNT7A* promoter upon pharmacological hypoxia, the time course of Wnt7a protein expression was analyzed. Data revealed that IOX2 pre-treatment induced a 1.4-, 1.7-, and 2-fold increase at 6, 12, and 24 h (Supplementary Figure 7A). Along this line, murine myoblasts pre-treated with FG-4592 showed a 1.55- and 1.7-fold increase at 12 and 24 h, respectively (Supplementary Figure 7B). Based on these results, the accumulation of Wnt7a protein induced by the pharmacological hypoxia was investigated at 24 h as compared to the untreated cells, revealing a 1.5- and 1.3-fold increase in murine myoblasts pre-treated with IOX2 and FG-4592, respectively (Figure 2G). To exclude that other Wnt proteins were induced by IOX2 and FG-4592 pre-treatment, *WNT1*, *WNT3a*, *WNT4*, *WNT9a*, and *WNT11* gene expressions were determined. Results revealed that murine myoblasts did not express the isoforms *WNT1* and *WNT3a*, and that pre-treatment with PHDs inhibitors did not induce any alterations in the gene expression of *WNT4*, *WNT9a*, and *WNT11* (Supplementary Figure 8).

### Treatment With PHDs Inhibitors Induces Myogenesis

**FIGURE 3:**
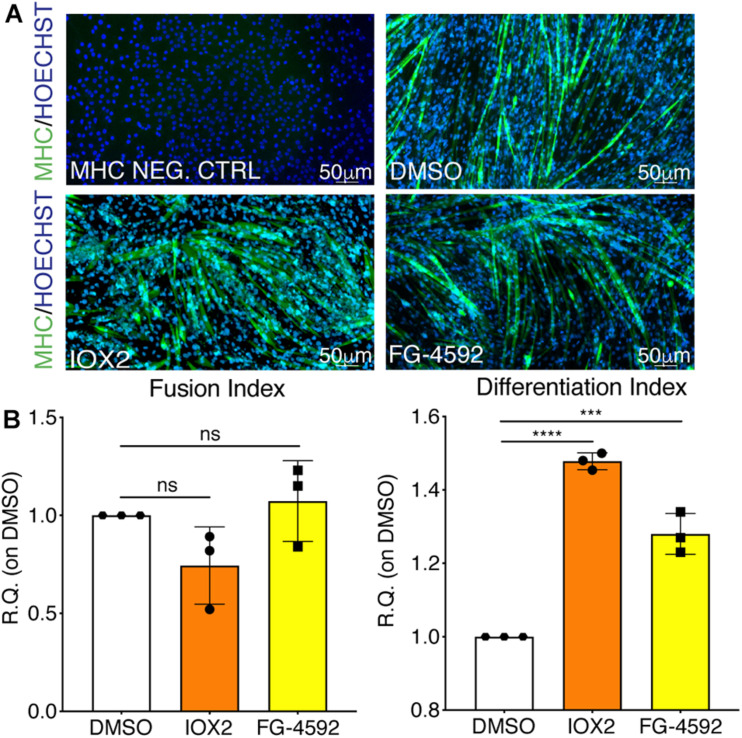
Effects of hypoxia inducible factor-1α (HIF-1α) pharmacological activation on skeletal muscle differentiation. **(A)** Immunofluorescence staining of myosin heavy chain (MHC; green) in proliferating (MHC NEG. CTRL) and differentiated murine myoblasts treated with IOX2 or FG-4592 for 24 h in growth medium (GM) and then switched to differentiation medium (DM) without the prolyl-hydroxylases (PHDs) inhibitors for 7 days. Nuclei were stained with Hoechst 33342. Magnification is 200×. **(B)** Quantification of the fusion index, as the ratio between MHC-positive nuclei and the total number of nuclei, and of the differentiation index, as the ratio between myotubes area and MHC-positive nuclei at the end of differentiation (*n* = 3). Data information: all data represent mean ± SD. ***p < 0.001, ****p < 0.0001 (Ordinary one-way ANOVA).

**FIGURE 4:**
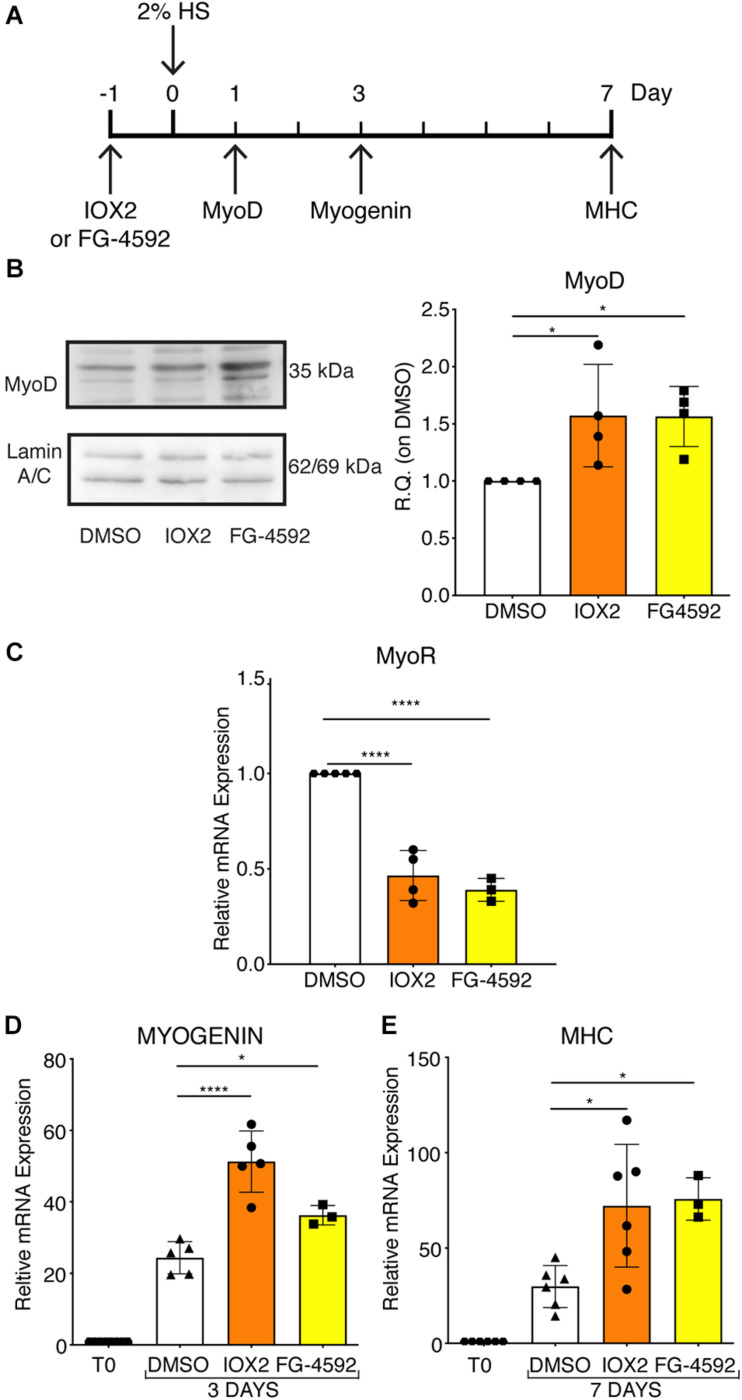
Modulation of myogenic differentiation markers upon pharmacological hypoxia. **(A)** Schematic representation of myogenic marker during skeletal muscle differentiation. **(B)** Western blot analysis and relative quantification of MyoD nuclear localization of murine myoblasts treated for 24 h with IOX2 or FG-4592. The nuclear marker Lamin A/C was used as the housekeeper (*n* = 4). **(C)** qPCR analysis of MyoR upon prolyl-hydroxylases (PHDs) inhibitors treatment (*n* = 4 for IOX2 and *n* = 3 for FG-4592). **(D,E)** qPCR analyses of MYOGENIN at 3 days (**D**, *n* = 5 for IOX2 and *n* = 3 for FG-4592) and of myosin heavy chain (MHC) at 7 days of differentiation (**E**, *n* = 6 for IOX2 and *n* = 3 for FG-4592), in murine myoblasts pre-treated with IOX2 or FG-4592. Data information: all data represent mean ± SD. *p < 0.05, ****p < 0.0001 (Ordinary one-way ANOVA).

To assess whether a pharmacological activation of HIF-1α would affect myogenesis, C2C12 murine myoblasts were pre-treated with IOX2 or FG-4592 in GM for 24 h, and then induced to differentiate for 7 days by switching them to DM without the PHDs inhibitors. At the end of the differentiation process, an extensive formation of MHC-positive myotubes was sighted in both pre-treated and untreated cells, whereas no signal was detected in the negative controls (Figure 3A). Quantitative evaluation of the differentiation parameters revealed no significant changes in the fusion indexes, whereas a 1.5- and 1.3-fold increase in the differentiation indexes could be observed in IOX2 and FG-4592 pre-treated myoblasts, respectively, as compared to untreated controls (Figure 3B). Next, the alterations of MyoD, Myogenin, and MHC, which are, respectively, the early, intermediate, and late myogenic differentiation markers, were investigated (Figure 4A). Protein expression analyses revealed that IOX2 and FG-4592 induced a 1.6- and 1.5-fold increase, respectively, of the nuclear localization of the early differentiation marker, MyoD (Figure 4B). Along this line, IOX2 and FG-4592 down-regulated the mRNA level of the main MyoD corepressor, *MYOR*, by 2.2- and 2.6-fold, respectively, compared to controls (Figure 4C). Finally, gene expression analyses showed a 2.0- and 1.4-fold increase of *MYOGENIN* at day 3 of differentiation, and a 2.2- and 2.3-fold enhancement of *MHC* at day 7 of differentiation, in IOX2 and FG-4592 pre-treated myoblasts, respectively (Figures 4D,E).

### *WNT7A* Silencing Counteracts the Effects of HIF-1α Activation on Myogenesis

**FIGURE 5:**
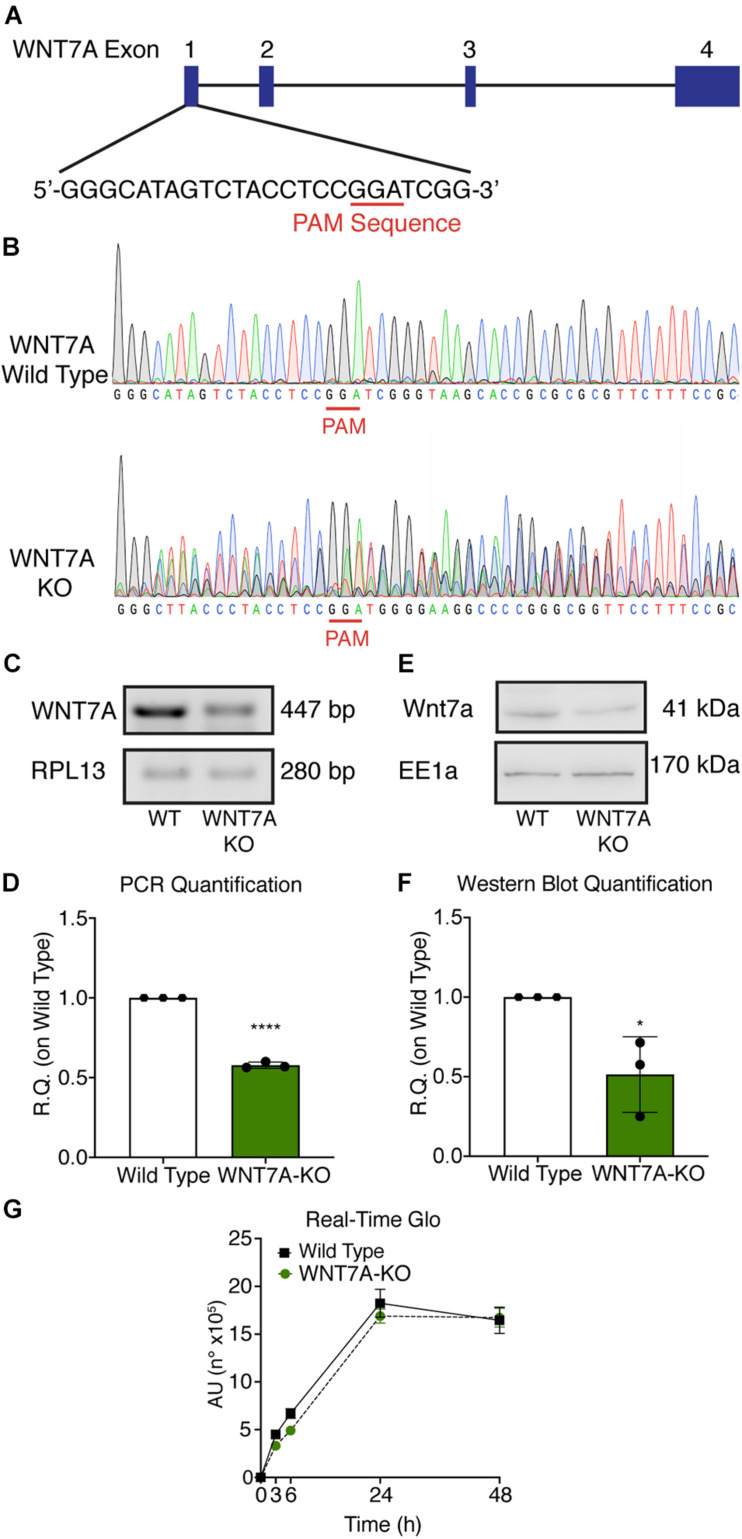
Characterization of *WNT7A* inhibition using CRISPR/Cas9 model in C2C12. **(A)** Graphical representation of the mouse *WNT7A*-targeted locus. Boxes and lines indicate exons and introns, respectively. The oligo represents sgRNA target sequence and Protospacer Adjacent Motifs (PAM) site is marked in red. **(B)** Sanger DNA sequencing were conducted on PCR products amplified from the genomic *WNT7A* loci of C2C12. **(C,D)** Genomic PCR analysis **(C)** and quantification of *WNT7A* inhibition **(D)**. RPLI was used as the housekeeper (*n* = 3). **(E,F)** Western Blot analysis **(E)** and quantification **(F)** of *WNT7A* expression in wild type (WT) and *WNT7A*-silenced murine myoblasts (*WNT7A*-KO; *n* = 3). **(G)** Cell viability analyzed by RealTime-Glo^TM^ kits MT Cell Viability Assay of wild type and *WNT7A*-silenced murine myoblasts (*n* = 3). Data information: all data represent mean ± SD. *p < 0.05, ****p < 0.0001 (Student’s *t*-test).

**FIGURE 6:**
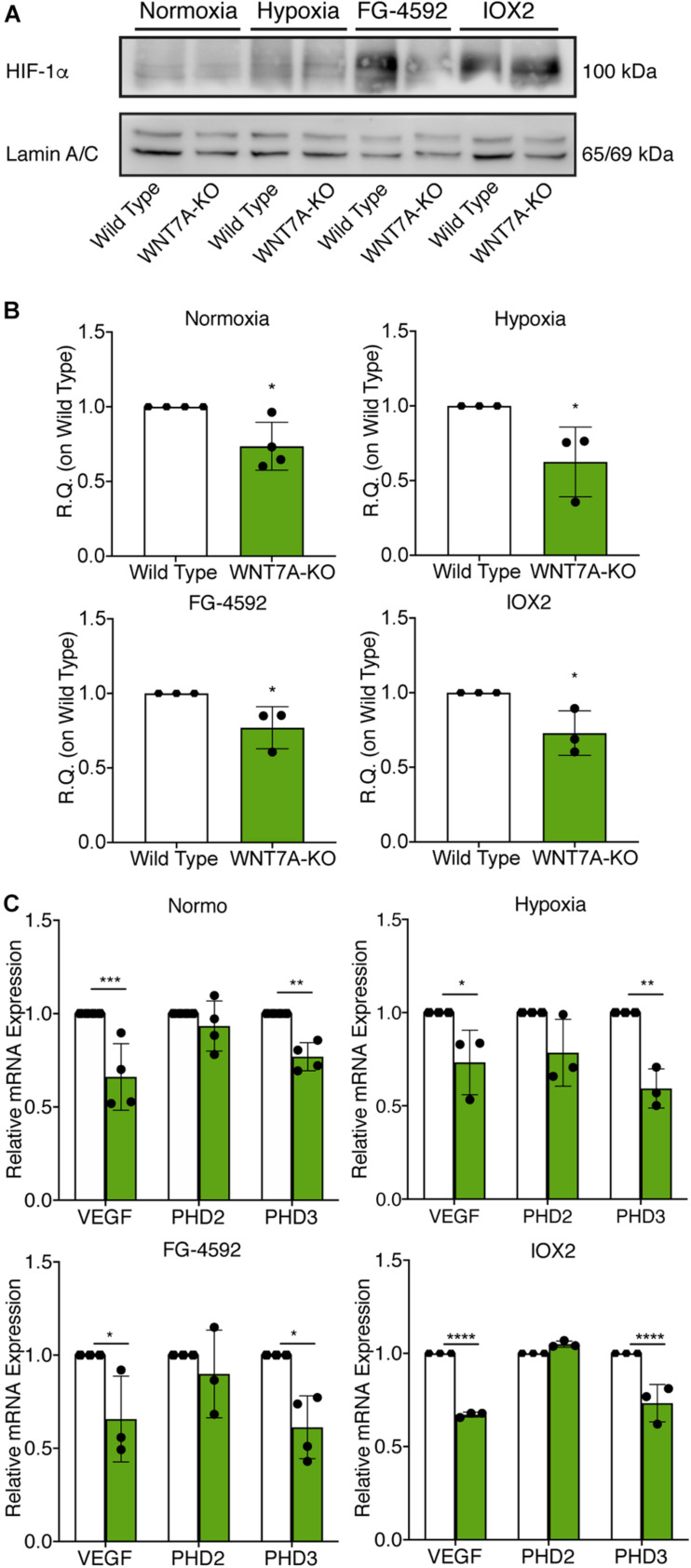
Modulation of hypoxia inducible factor-1α (HIF-1α) pathway induced by *WNT7A* silencing upon physical and pharmacological hypoxia. **(A,B)** Western blot analysis (A) and relative quantification (B) of HIF-1α nuclear localization in wild type (WT) and *WNT7A*-silenced murine myoblasts (*WNT7A*-KO) treated under normoxia, hypoxia, FG-4592 or IOX2. The nuclear marker Lamin A/C was used as the housekeeper (*n* = 4 for Normoxia, *n* = 3 for Hypoxia, IOX2 and FG-4592). **(C)** qPCR analysis of HIF-1α target genes, *VEGF*, *PHD2*, and *PHD3* in WT and WNT7A-silenced murine myoblasts (WNT7A-KO) upon physical and pharmacological hypoxia (*n* = 4 for Normoxia, *n* = 3 for Hypoxia, IOX2 and FG-4592). Data information: all data represent mean ± SD. For western blot analysis of HIF-1α *p < 0.05 (Student’s *t*-test). For qPCR analysis of HIF-1α target genes *p < 0.05, **p < 0.01, ***p < 0.001, ****p < 0.0001 (ordinary one-way ANOVA).

**FIGURE 7:**
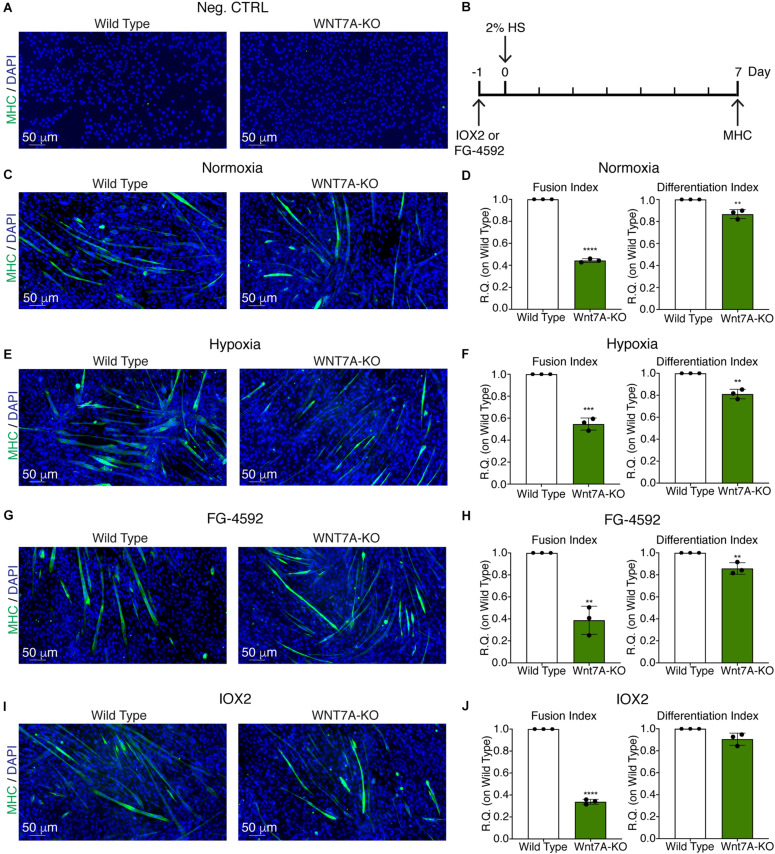
Effects of *WNT7A* inhibition on skeletal muscle differentiation upon physical and pharmacological hypoxia. **(A)** Negative control of myosin heavy chain (MHC) staining in proliferation wild type (WT) and *WNT7A*-silenced murine myoblasts (*WNT7A*-KO)**. (B)** Schematic representation of MHC staining at 7 days of the differentiation process. **(C,E,G, I)** Immunofluorescence staining of MHC (green) in WT and *WNT7A*-silenced murine myoblasts (*WNT7A*-KO) treated under normoxia **(C)**, hypoxia **(E)**, FG-4592 **(G),** or IOX2 **(I)** for 24 h in GM and then switched to differentiation medium (DM) without the prolyl-hydroxylases (PHDs) inhibitors for 7 days. Nuclei were stained with Hoechst 33342. Magnification is 200× (*n* = 3). **(D,F,H,J)** Quantification of the fusion index, as the ratio between MHC-positive nuclei and the total number of nuclei, and of the differentiation index, as the ratio between myotubes area and MHC-positive nuclei at the end of differentiation (*n* = 3). Data information: all data represent mean ± SD. **p < 0.01, ***p < 0.001, ****p < 0.0001 (Student’s *t*-test).

**FIGURE 8:**
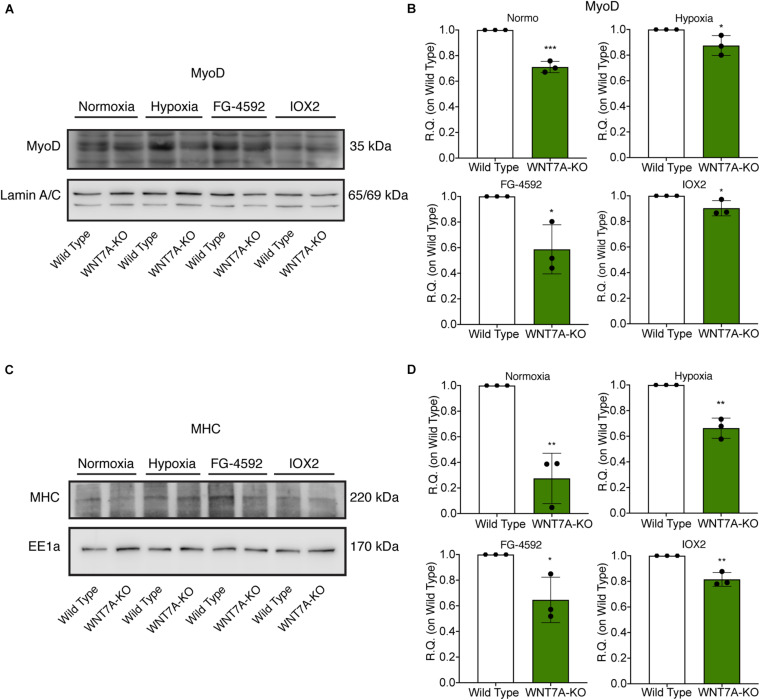
Modulation of MyoD nuclear translocation and myosin heavy chain (MHC) expression in wild type (WT) and *WNT7A*-silenced murine myoblasts upon physical and pharmacological hypoxia. **(A,B)** Western blot analysis **(A)** and relative quantification **(B)** of MyoD nuclear localization were tested after 24 h preconditioning under physical or chemical hypoxia in WT and *WNT7A*-KO. The nuclear marker Lamin A/C was used as the housekeeper (*n* = 3). **(C,D)** Western blot analysis **(C)** and relative quantification **(D)** of MHC were measured at the end of the skeletal muscle differentiation in WT and *WNT7A*-KO. The early endosomal antigen 1a (EE1a) was used as the housekeeper (*n* = 3). Data information: all data represent mean ± SD. *p < 0.05, **p < 0.01, ****p < 0.0001 (Student’s *t*-test).

To further elucidate the direct involvement of Wnt7a in mediating HIF-1α effects on myogenesis, *WNT7A* silencing was performed using the CRISPR/Cas9 genome editing in C2C12 murine myoblasts. To this purpose, a gRNA sequence targeted into exon 1, of the four present in the *WNT7A* gene, was selected (Figure 5A). A non-targeting gRNA sequence, which is unable to recognize any sequence in the human genome, was used as a negative control. PCR products of the *WNT7A*-targeted genomic region were analyzed by Sanger sequencing, confirming the introduction of a frameshift in the gene (Figure 5B). To quantify *WNT7A* silencing, quantitative PCR and Western Blot analysis were performed on wild-type and *WNT7A*-KO myoblasts. In particular, results demonstrated that *WNT7A*-KO cells exhibited a 44% decrease in *WNT7A* genomic expression (Figures 5C,D) and a 49% reduction of Wnt7a total protein (Figures 5E,F), as compared to wild-type cells. After confirming the *WNT7A* silencing, the analysis of cell proliferation was performed revealing that *WNT7A*-KO cells did not exhibit significant modifications in cell growth rate, as compared to the wild type cells (Figure 5G). Next, the effects of *WNT7A* silencing on the activation of the HIF-1α pathway, upon physical or pharmacological hypoxia, were investigated (Figure 6A). In particular, *WNT7A-KO* cells showed a 27% reduction of HIF-1α nuclear localization under normoxia (Figure 6B). The decrease of HIF-1α into the nuclei was also confirmed following physical and pharmacological hypoxia. Indeed, *WNT7A*-KO cells showed a 38%, 24%, and 28% decrease in HIF-1α nuclear localization when *WNT7A*-KO cells were cultured under hypoxia (1% oxygen) or with IOX2 and FG-4592, respectively (Figure 6B). Moreover, reduction of HIF-1α into the nuclei reflected a significant down-regulation of its main target genes. In particular, *WNT7A*-KO cells showed a 36% and 24% decrease in *VEGF* and *PHD3* gene expression, whereas no statistical significance was observed in the gene expression of *PHD2* under normoxia culture condition (Figure 6C). Similarly, silencing of *WNT7A* induced a 27% and 41% reduction in the gene expression of *VEGF* and *PHD3*, respectively, under physical hypoxia (Figure 6C). Similarly, pre-treatment with FG-4592 and IOX2 reduced by 39% and 27% the gene expression of *PHD3*, respectively, and by 33% that of *VEGF* (Figure 6C). Then, the effects of *WNT7A* silencing were assessed on myogenesis at the end of the differentiation process, ultimately evaluating the formation of MHC-positive myotubes, which was undetectable in the negative control of wild type and *WNT7A*-KO cells (Figures 7A,B). Then, *WNT7A*-KO cells were then induced to differentiate under normoxic conditions for 7 days showing a 56% and a 15% reduction in the fusion and differentiation indexes as compared to wild-type controls, respectively (Figures 7C,D). Then, we assessed whether *WNT7A* silencing would hamper the beneficial effects on myogenesis of HIF-1α induced-activation. To this purpose, *WNT7A*-KO cells were subjected to hypoxic (1% oxygen) conditions or incubated with PHDs inhibitors FG-4592 or IOX2 to induce HIF-1α activation before differentiation under normoxia. Results showed that *WNT7A*-KO cells exhibited a 46% and 20% reduction in the fusion and differentiation indexes, respectively, when subjected to a pre-treatment under physical hypoxia (Figures 7E,F). Similarly, *WNT7A*-KO cells showed a 63% and 67% decrease in the fusion indexes upon FG-4592 and IOX2 pre-treatment, respectively (Figures 7G–J). Moreover, a 15% reduction in the differentiation index was induced by FG-4592 pre-treatment, while no significant changes were observed with IOX2 (Figures 7G–J). To further assess whether *WNT7A* silencing would hinder the effects of HIF-1α activation, MyoD and MHC protein expression was determined. Results showed that MyoD nuclear localization decreased by 29%, 13%, and 42% under normoxia, hypoxia, and FG-4592 pre-treatment conditions, respectively, while no significant changes were observed with IOX2, as compared to WT cells (Figures 8A,B). Similarly, *WNT7A*-KO cells exhibited a marked down-regulation of MHC expression at the end of the differentiation process, as MHC decreased by 80%, 34%, 36%, and 18% under normoxic, hypoxic, FG-4592, and IOX2 pre-treatment conditions, respectively (Figures 8C,D).

### Effects of FG-4592 Injection on Skeletal Muscle Regeneration *in vivo*

**FIGURE 9:**
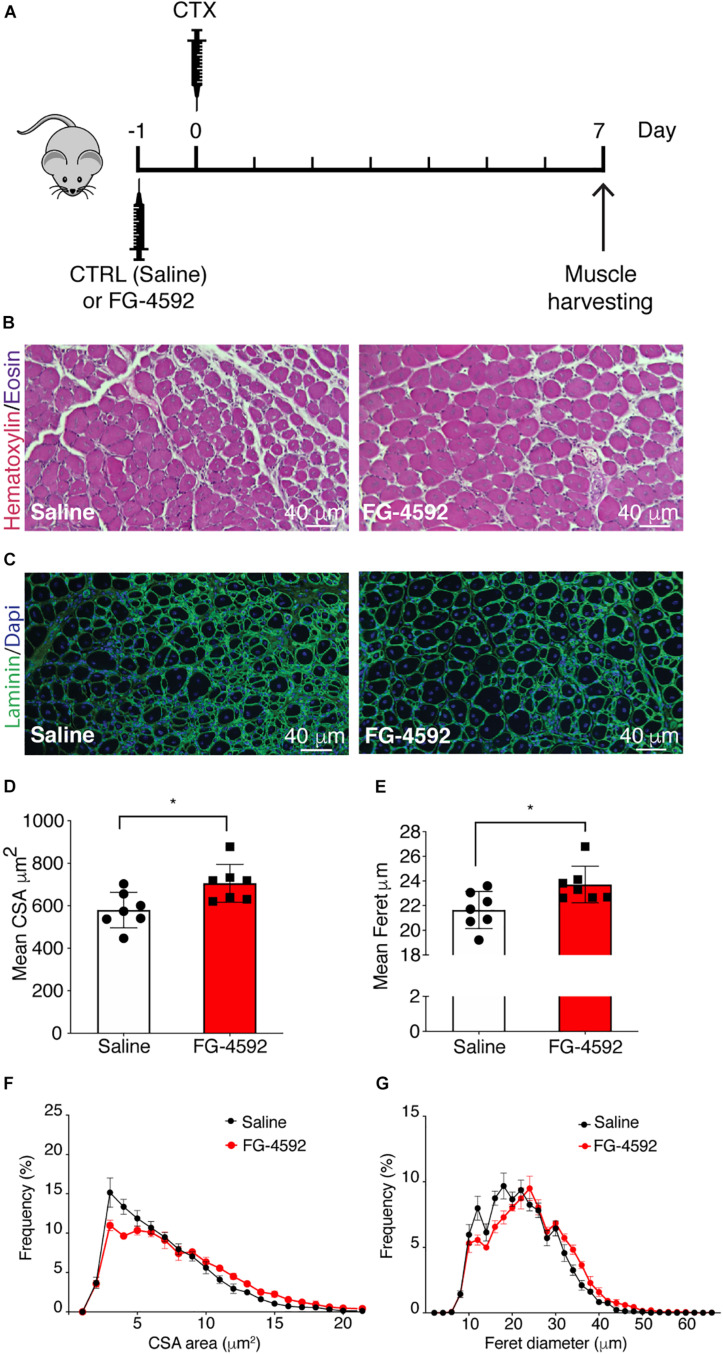
Effects of FG-4592 treatment on muscle regeneration. **(A)** Schematic illustration of the experimental procedure: 24 h before cardiotoxin (CTX) injury of tibialis anterior (TA) muscle, mice were injected by i.p. with FG-4592 (10 mg/kg). Mice were sacrificed 7 days after CTX-induced injury, and TA muscles were collected. **(B)** Immunohistochemical detection of hematoxylin/eosin in FG-4592 pre-treated mice, compared to controls. Scale bars = 40 μM (*n* = 7 mice for each group). **(C)** Immunofluorescence staining of laminin (green) in saline and FG-4,592 pre-treated mice. Nuclei were stained with Hoechst 33,342. Scale bars = 40 μM (*n* = 7 mice for each group). **(D,E)** Quantification of CSA **(D)** and minimal Feret diameter **(E)** averages in FG-4592 or saline pre-treated mice. **(F,G)** Representation of CSA **(F)** and minimal Feret diameter **(G)** distribution in saline or FG-4592 pre-treated mice. Data information: all data represent mean ± SD. *p < 0.05 (Student’s *t*-test).

**FIGURE 10:**
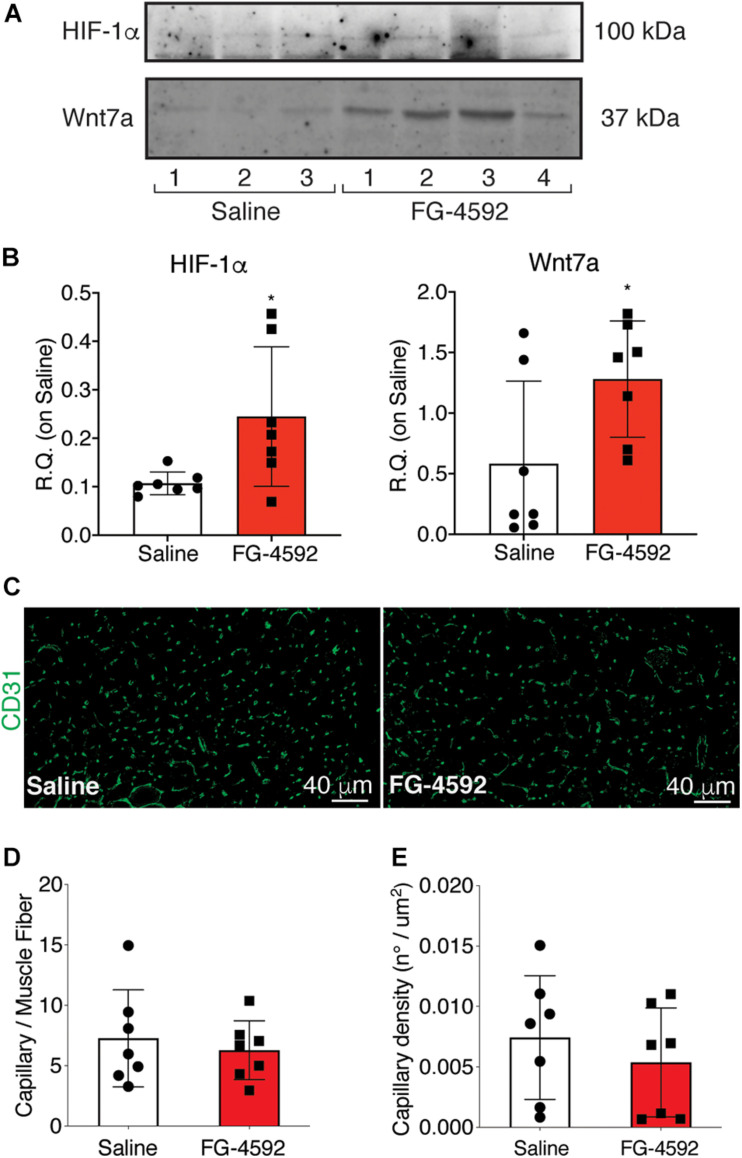
Assessment of activation of hypoxia inducible factor-1α (HIF-1α) pathway in FG-4,592 or saline treated mice upon cardiotoxin CTX-induced muscle injury. **(A,B)** Western blot analysis (A) and relative quantification (B) of HIF-1α and Wnt7a accumulation in saline and FG-4592 pre-treated mice 7 days after CTX-induced muscle injury (*n* = 7). **(C)** Immunofluorescence staining of CD31 (green) on tibial anterior muscle sections of saline and FG-4592 pre-treated mice 7 days after CTX-induced muscle injury (Scale bars = 40 μM). **(D** and **E)** Quantification of the total number of capillaries (D), normalized for the total number of muscle fibers, and the density of capillaries (E), calculated as the total number of capillaries on the area of the fibers, in saline or FG-4592 pre-treated mice (*n* = 7 for each group). Data information: all data represent mean ± SD. *p* > 0.05, n.s., *p < 0.05 (Student’s *t*-test).

To assess whether a pharmacological activation of HIF-1α would affect skeletal muscle regeneration *in vivo*, C57Bl/6N mice were pre-treated with a single intraperitoneal injection of saline solutions or FG-4592 (10 mg/kg, as previously reported as optimal to induce HIF-1α activation in mice, and comparable to a 40–50 μM concentration in the *in vitro* experiments), 1 day before CTX-induced degeneration of the TA muscles (Figure 9A; Xuan et al., 2018). FG-4592 pre-treated mice were sacrificed 7 days after injury and, while Hematoxylin/Eosin analyses revealed no macroscopic difference between FG-4592-injected and control muscles (Figure 9B), laminin staining showed a 25% enhancement of cross-sectional area (CSA) and an 8% increase of minimal Feret’s diameter of regenerating myofibers, characterized by centrally located nuclei (Figures 9C–E). These results were also confirmed by CSA and minimal Feret’s diameter distribution analyses, proving that FG-4592 injection induced a shift of a frequency distribution toward larger fibers (Figures 9F,G). Then, to confirm that pre-treatment with FG-4592 promoted muscle regeneration by the activation of HIF-1α pathway, the total amount of HIF-1α and Wnt7a proteins were analyzed revealing a 2.2- and a 2.4-fold increase, respectively (Figures 10A,B). Finally, to exclude the possibility that the effects on muscle regeneration could be due to the activation of angiogenesis induced by FG-4592, CD31 staining was performed on the muscle sections (Figure 10C). Results indicated that FG-4592 treatment did not significantly modify the number of regenerating fibers nor the density of capillaries at 7 days after the muscle injury, compared to control mice (Figures 10D,E), supporting the hypothesis that FG-4592 promotes muscle regeneration independently from angiogenesis activation.

## Discussion

**FIGURE 11:**
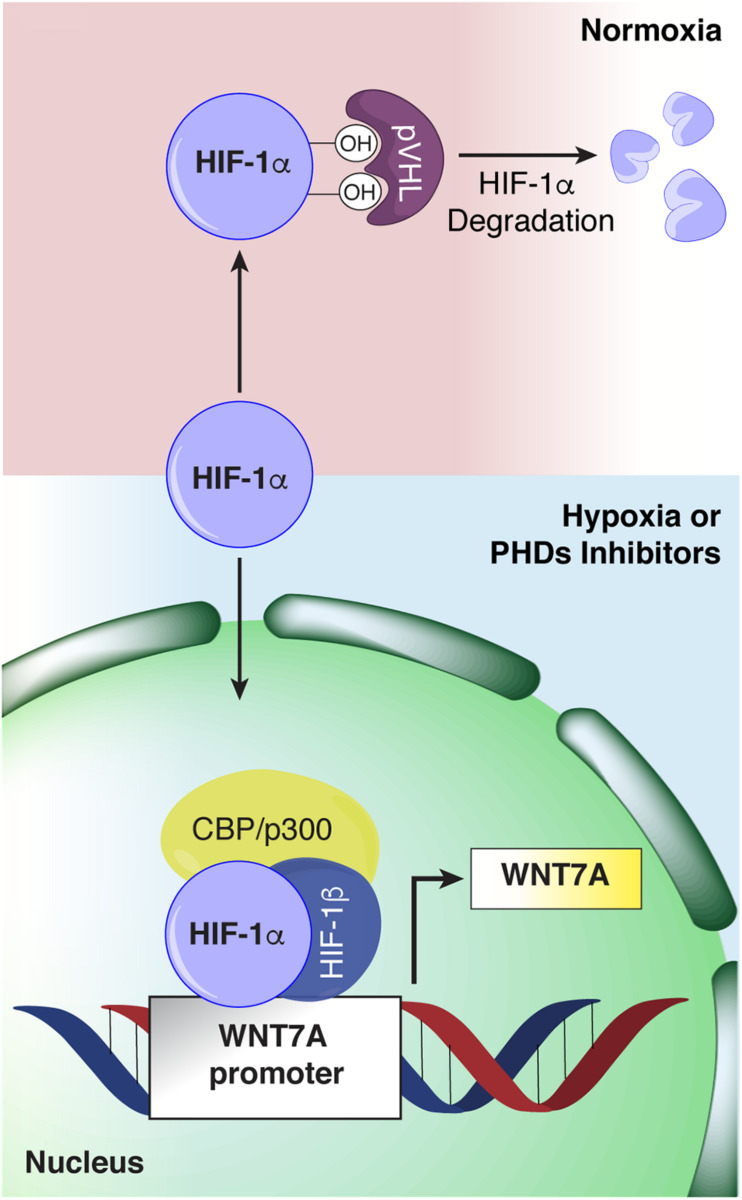
Schematic representation of hypoxia inducible factor-1α (HIF-1α) binding to the *WNT7A* promoter. Under normoxia, HIF-1α is hydroxylated by the active prolyl-hydroxylases (PHDs) and sent to proteasomal degradation. Under hypoxia or upon PHDs inhibition, HIF-1α cannot be hydroxylated, and it translocates to the cell nucleus, where it forms a transcriptional complex with its α-subunit and CBP/p300, and binds to specific HRE sequences on the *WNT7A* promoter region.

The discovery that HIF-1α regulates *WNT7A* gene expression in skeletal muscle cells by directly binding to its promoter (Figure 11) unveils a novel mechanism in the complex myogenic machinery. Remarkably, this could lead to the development of new therapeutic approaches for muscular diseases. While the key role of Wnt7a in muscle regeneration has been established, attempts to therapeutically exploit it have faced the drawback of the high hydrophobicity of its protein (von Maltzahn et al., 2013). On the other hand, a pharmacological activation of HIF-1α has been shown to be feasible, also in humans, and several clinical trials for non-muscular pathologies, including renal anemia, are currently in advanced phases (von Maltzahn et al., 2013; Maxwell and Eckardt, 2016). In particular, HIF is a transcription factor constituted by HIF-1β which is constitutively expressed, and HIF-1α or HIF-2α, which are the oxygen-sensitive subunits (Wang et al., 1995). At high oxygen concentration, the α-subunit is recognized and hydroxylated by the PHDs, which use oxygen and 2-oxoglutarate (2-OG) as co-substrates, promoting its proteasomal degradation (Kim and Kaelin, 2003). Otherwise at low oxygen levels, the PHDs are inhibited, and HIF-1α can translocate to the nuclei and induce the expression of its target genes (Kim and Kaelin, 2003). In this contest, we also reported a novel, PHD independent, activation of HIF-1α by sialidase NEU3, which may also be further exploited in the future (Scaringi et al., 2013; Piccoli et al., 2017). HIF-1α activation has been shown to promote neo-angiogenesis and cell survival in several pathologies, including cancer (Krock et al., 2011; Hubbi and Semenza, 2015). However, none of the previous studies recognized its direct contribution to the myogenic machinery and even created the misconception that HIF-1α activation would primarily impair the process (Di Carlo et al., 2004; Majmundar et al., 2015). Ultimately, the current notion is that the HIFs, which are induced under the hypoxic conditions typical of satellite cell niches, contribute to preserving muscle progenitor cells in a quiescent, undifferentiated state (Di Carlo et al., 2004; Gustafsson et al., 2005; Clarke and van der Kooy, 2009; Eliasson and Jonsson, 2010; Mohyeldin et al., 2010). Along this line, Di Carlo et al. (2004) reported the first evidence that exposure to prolonged hypoxia blocks myogenesis, and inhibited MyoD, Myf5, and MYOGENIN expression. More recently, it was shown that hypoxic culture conditions favor the quiescence of satellite cell-derived primary myoblasts by upregulating Pax7, a key regulator of satellite cell self-renewal, and downregulating MyoD and Myogenin (Liu et al., 2012). Overall, these and other studies corroborated the notion that hypoxia is detrimental for muscle differentiation, and HIFs activation was acknowledged to inhibit the process (Majmundar et al., 2015). Actually, the role of each of the three different HIFs (HIF-1α, HIF-2α, and HIF-3α) has been shown to be distinct, and their activation is dependent on the degree and the duration of oxygen deprivation (Wang et al., 1995; Dengler et al., 2014; Yang et al., 2017). In particular, it was established that, among all the different HIFs, only HIF-2α is responsible for maintaining satellite cells in a quiescent state (Xie et al., 2018). HIF-2α is activated under mild hypoxia (1.3%, consistent with the oxygen tension of the stem cell niche), whereas HIF-1α appears to be induced only under extreme hypoxia (below 1% O_2_), such as in the case of an ischemic event that causes muscle damage followed by regeneration (Yang et al., 2017; Xie et al., 2018). In this context, our study unveils that a 24 h pre-conditioning under hypoxia (1% oxygen) did not induce the activation of HIF-2α, although we cannot rule out its involvement at longer exposure times, whereas it promoted the nuclear translocation of HIF-1α, which in turn activated myogenesis. In particular, we discovered that HIF-1α directly binds two HREs on the *WNT7A* promoter, ultimately activating muscle regeneration. At this stage, we cannot exclude that *WNT7A* could be a general target of HIF-1α also in other cells, as the binding was also observed in a model of oligodendrocytes (Yuen et al., 2014).

Based on these results, we investigated the possibility of pharmacologically induce *WNT7A*, and ultimately myogenesis, by administering some PHDs inhibitors, which are known as HIF-1α activators. In particular, we assessed the effects of IOX2 and FG-4592 (the latter also known as Roxadustat^®^), two commercially available PHDs inhibitors, which present higher selectivity for PHD2 as compared to other 2-OG dioxygenases (Chiang Chan et al., 2015; Yeh et al., 2017). Remarkably, both compounds induced myogenesis *in vitro* and promoted myotube hypertrophy. Interestingly, treatment of skeletal myoblasts with PHDs inhibitors activated MyoD nuclear translocation even before subjecting cells to differentiation, supporting the hypothesis that the drug increased their commitment to myogenesis. The treatment also caused an early and increased expression of the differentiation markers Myogenin and MHC, similarly to what it was previously observed with Wnt7a induction or supplementation (von Maltzahn et al., 2011). These effects were reverted by *WNT7A* silencing *via* CRISPR/Cas9 genome editing. Actually, we could only obtain a 44% reduction of *WNT7A*, possibly due to the presence of a contamination of untransfected cells. Nonetheless, this reduction was sufficient to significantly impair myogenesis.

Overall, these results support the critical role played by Wnt7a in the activation of myogenesis upon a hypoxic stimulus. In particular, the HRE binding sequences on the *WNT7*A promoter suggest that the activation of HIF-1α, which has been observed after a hypoxic damage in a muscle, is instrumental for activating myoblasts to regenerate the tissue. Thus, we tested this hypothesis also *in vivo* in a mouse model of CTX-induced injury. Mice were pre-treated with a single intraperitoneal injection of 10mg/kg FG-4592, which has already been shown to activate the HIF-1α pathway without evident toxic effects in mice models and is comparable to the dose used for the *in vitro* assays (Xuan et al., 2018). Results showed that FG-4592 treatment induced the accumulation of HIF-1α and Wnt7a in the TA muscles, eventually improving muscle regeneration after CTX-induced muscle injury and leading to the formation of significantly larger fibers. Notably, we demonstrated that the effects were independent from an activation of angiogenesis, which could also be induced by HIF-1α, as no changes in the endothelial marker CD31 expression could be observed (Razban et al., 2012). Based on these results, a post-conditioning model of muscle injury could unveil the potential of this (or other) PHDs inhibitors in counteracting several skeletal muscle diseases and in developing of a new therapeutic approach characterized by multiple injections or by other routes of drug administration. Along this line, it is reasonable to hypothesize a possible role of HIF-1α in skeletal muscle atrophy in the elderly. Indeed, aging is a physiological condition which shows a metabolic shift of myofibers from glycolytic to oxidative metabolism (Ohlendieck, 2011). The increase of the oxidative myofibers could be due to a reduction of HIF-1α activation and the expression of its target genes (Higashimura et al., 2011). Based on these premises, it would be interesting to test the effects of these PHDs inhibitors in pathologies characterized by skeletal muscle atrophy as primary or secondary effects, such as cancer, Acquired Immunodeficiency Syndrome (AIDS), diabetes, and heart failure. Studies in this direction are ongoing in our laboratory.

## Data Availability Statement

All datasets generated in this study are included in the article/Supplementary Material Table 1.

## Ethics Statement

The animal study was reviewed and approved by Italian Ministry of Health.

## Author Contributions

LA and FC conceived the study and designed and analyzed all the experiments. LA, FC, MF, CP, EA, MM, GC, and AGr wrote the manuscript. FC, GR, AT, PR, MP, and AGh performed all the *in vitro* experiments. MF and EA performed all the *in vivo* study. LA, FC, and MP prepared the figures. All authors reviewed the results and approved the final version of the manuscript.

## Conflict of Interest

The authors declare that the research was conducted in the absence of any commercial or financial relationships that could be construed as a potential conflict of interest.

## Footnotes

1 www.stat.uiowa.edu/∼rlenth/Power/index.html

2 http://www.latoxan.net/
