# Supplementary material for: HIF-1α Directly Controls WNT7A Expression During Myogenesis
Source: Front Cell Dev Biol. 2020 Nov 11;8:593508. doi: 10.3389/fcell.2020.593508 (PMC7686515; doi:10.3389/fcell.2020.593508)
Supplement: Supplementary file 2 [file Table_2.docx]

Supplementary Material

# Supplementary Figure 1

**Supplementary Figure 1 - Characterization of *WNT7A* promoter activity in HIF-1α-silenced myoblasts. (A, B)** Western blot analysis (A) and relative quantification (B) of HIF-1α nuclear translocation in HIF-1α-silenced (shHIF-1α) and wild-type (HIF-1α) murine myoblasts by Western Blot. The nuclear marker Lamin A/C was used as the housekeeper (n=3). **(C)** Schematic representation of pNL1.1 plasmid containing the *WNT7A* promoter upstream of the luciferase gene reporter. **(D)** Quantification of pNL1.1- *WNT7A* promoter activity by luciferase assay in HIF-1α-silenced (shHIF-1α), as compared to wild-type cells (HIF-1α) (n=5). Data information: all data represent mean ± SD. *p<0.05, **p<0.01 (Student’s t-test).

# Supplementary Figure 2

**Supplementary Figure 2- Positive control of ChIP assay. (A)** Schematic representation of the known HRE recognized by HIF-1α on the *VEGF* promoter, which was used as a positive antibody control for the ChIP assay. **(B)** qPCR of HRE on VEGF promoter recognized and bound by HIF-1α upon hypoxic preconditioning (n=3). Data information: all data represent mean ± SD. *p < 0.05, **p<0.01 (Ordinary one-way ANOVA)

**Supplementary Figure 3**

**Supplementary Figure 3- Sequences of *WNT7A* promoter not involved in HIF-1α binding**. **(A)** Schematic representation of the *WNT7A* promoter divided in seven binding sequences amplified by qPCR after Chromatin Immunoprecipitation. **(B)** Real-time Quantitative PCR of Seq.2, Seq.3, Seq.5, Seq.6 and Seq.7 not implicated in the binding of HIF-1α on the *WNT7A* promoter (n=3). Data information: all data represent mean ± SD. p > 0.05 n.s. (Ordinary one-way ANOVA).

**Supplementary Figure 4**

**Supplementary Figure 4 – Time course of Wnt7a protein upon normoxia and hypoxia. (A, B)** Western blot analysis and relative quantification of Wnt7a protein in murine myoblasts treated for 3, 6, 12 and 24 h under normoxia(A) or hypoxia (B) culture conditions. The marker EE1a was used as the housekeeper (n=3). Data information: all data represent mean ± SD. n.s. p>0.05, *p < 0.05, (Ordinary one-way ANOVA).

**Supplementary Figure 5**

**Supplementary Figure 5 – Assessment of HIF-2α nuclear translocation. (A, B)** Western blot analysis (A) and relative quantification (B) of HIF-2α nuclear translocation in murine myoblasts treated for 24 h under normoxia and hypoxia (1% O_2_). The nuclear marker Lamin A/C was used as the housekeeper (n=3). Data information: all data represent mean ± SD. n.s. p> 0.05, (Student’s t-test).

**Supplementary Figure 6**

**Supplementary Figure 6 - Analysis of murine myoblasts viability upon PHDs chemical treatment. (A)** Cell viability analyzed by RealTime-Glo™ kits MT Cell Viability Assay (n=3). **(B)** Cell toxicity measured with CellTox™ Cytotoxicity Assay (n=3). Data information: all data represent mean ± SD. n.s. p > 0.05 (Ordinary one-way ANOVA).

**Supplementary Figure 7**

**Supplementary Figure 7 – Time course of Wnt7a protein upon IOX2 and FG-4592 pre-treatment. (A, B)** Western blot analysis and relative quantification of Wnt7a protein in murine myoblasts treated with IOX2 (A) or FG-4592 (B) for 3, 6, 12 and 24 h. The marker EE1a was used as the housekeeper (n=3). Data information: all data represent mean ± SD. *p < 0.05, **p<0.01, ****p<0.0001(Ordinary one-way ANOVA).

**Supplementary Figure 8**

**Supplementary Figure 8- Effects of a pharmacological activation of HIF-1α on WNT genes expression.** qPCR analysis of WNT4, WNT9a and WNT11 genes in murine myoblasts treated for 24 h with IOX2 or FG-4592 (n=3).

Data information: all data represent mean ± SD. *p < 0.05, ***p<0.001 (Ordinary one-way ANOVA).
